# *Weissella confusa* RY-1 ameliorates DSS-induced colitis by restoring intestinal barrier, modulating gut microbiota, and reprogramming microbial metabolism

**DOI:** 10.3389/fmicb.2026.1896924

**Published:** 2026-08-05

**Authors:** Huaqin Hu, Xin Yao, Huan Zhou, Lu Wang, Bin Peng, Changjin Liu, Shilu Luo, Jian Huang, Xun Min

**Affiliations:** 1 Department of Laboratory Medicine, Affiliated Hospital of Zunyi Medical University, Zunyi, Guizhou, China; 2 School of Laboratory Medicine, Zunyi Medical University, Zunyi, Guizhou, China

**Keywords:** intestinal barrier, intestinal flora, metabolism, ulcerative colitis, *W. confusa* RY-1

## Abstract

Ulcerative colitis (UC) remains a challenging chronic inflammatory bowel disease with limited treatment options. This study investigates the protective role of *Weissella confusa* RY-1, a potential probiotic strain isolated from human gastric fluid, in a dextran sulfate sodium (DSS)-induced murine colitis model. Administration of *W. confusa* RY-1 significantly ameliorated colitis symptoms in mice subjected to a 10-day colitis induction model, as demonstrated by improved body weight recovery, reduced disease activity index (DAI) scores, attenuated colon shortening, and restored colonic histoarchitecture. The probiotic treatment enhanced intestinal barrier integrity through upregulation of tight junction proteins ZO-1 and Occludin. 16S rRNA sequencing revealed substantial modulation of gut microbiota, characterized by reduced abundance of pro-inflammatory *Escherichia-Shigella* and enrichment of beneficial *Akkermansia*. Metabolomic analysis further demonstrated metabolic reshaping with elevated anti-inflammatory linoleic acid and decreased succinic acid levels. These findings collectively indicate that *W. confusa* RY-1 exerts protective effects against colitis through multiple mechanisms including barrier reinforcement, microbiota modulation, and metabolic reprogramming, suggesting its promising potential as a novel probiotic-based intervention for ulcerative colitis.

## 1 Introduction

The Ulcerative colitis (UC) is a chronic, non-specific inflammatory bowel disease characterized by persistent and diffuse inflammation of the colonic mucosa and submucosa (Niu et al., 2021). Although its exact etiology remains unclear, UC is widely believed to arise from a complex interplay of genetic predisposition, immune dysregulation, environmental factors, and alterations in the gut microbiota (Xu et al., 2023). In recent years, the incidence and prevalence of UC have continued to increase, particularly among individuals aged 30–40, imposing a substantial burden on global healthcare systems (Le Berre et al., 2023). UC is widely recognized as a refractory and relapsing inflammatory disorder of the gastrointestinal tract due to its diverse clinical manifestations and complex pathology, which considerably complicate treatment (Dong et al., 2022). Current therapeutic strategies primarily rely on anti-inflammatory agents, corticosteroids, and immunomodulators, which aim to control symptoms rather than achieve a cure (Guo and Lv, 2023). Thus, there is an urgent need to explore novel and effective treatments for UC.

In this context, probiotics have emerged as a promising therapeutic option for UC and other gut-related disorders. Probiotics are defined as live microorganisms that confer health benefits to the host by modulating the intestinal microbiota, enhancing barrier function, and regulating immune responses (Quigley, 2019; Mafe et al., 2025). For instance, previous studies have demonstrated that *Lactobacillus plantarum* ZJ316 alleviates UC in a mouse model by reducing inflammation, modulating short-chain fatty acid levels, and restoring gut microbial balance (Gu et al., 2023). Similarly, *Bifidobacterium bifidum* CCFM681 has been shown to protect the intestinal barrier, modulate microbiota composition, and inhibit pro-inflammatory pathways, thereby ameliorating colitis (Chen et al., 2021). These findings underscore the potential of probiotics as an effective therapeutic strategy for UC.

Among various probiotic candidates, *W. confusa* has attracted attention due to its beneficial properties. Studies have confirmed that *W. confusa* produces antimicrobial substances such as bacteriocins and organic acids, which play a pivotal role in inhibiting the growth of pathogenic bacteria (Tenea and Lara, 2019; Goh and Philip, 2015; Sartono et al., 2019). For example, *W. confusa* DD_A7 was shown to reduce oxidative stress and inflammatory responses in a zebrafish model infected with *E. coli* O157: H7 (Dey and Kang, 2020), while *W. confusa* CGMCC 19308 enhanced host defense against *Salmonella typhimurium* (Wang W. et al., 2022). These studies highlight the potential of *W. confusa* as a valuable probiotic strain in alleviating inflammation and modulating gut health. Although one study indicated that *W. confusa* can mitigate inflammatory damage in a UC model (Sujaya et al., 2023), its precise mechanisms of action remain unclear. Moreover, the inter-regulatory relationships between the gut microbiota and intestinal barrier function in UC mice treated with *W. confusa* have not been sufficiently investigated. Elucidating these interactions could not only enhance our understanding of UC pathogenesis but also provide a theoretical foundation and clinical guidance for future research.

Previously, we isolated and identified *W. confusa* RY-1 from the gastric fluid of a healthy individual. Subsequent evaluations revealed promising probiotic properties and a favorable safety profile (Hu et al., 2025). Therefore, this study aimed to investigate how *W. confusa* RY-1 improves the intestinal ecosystem in a mouse model of UC—specifically through modulation of intestinal barrier function, microbial diversity and composition, and gut metabolite profiles—to ultimately alleviate DSS-induced colitis symptoms.

## 2 Materials and methods

### 2.1 Determination of pH in the supernatant of *W. confusa* RY-1

*W. confusa* RY-1 was isolated from the gastric juice of a healthy subject during a routine health examination. The strain was identified by sequencing and deposited at the China General Microbiological Culture Collection Center (CGMCC) under the accession number CGMCC No. 28783. The strain was first inoculated onto De Man, Rogosa and Sharpe (MRS) solid medium and incubated at 37 °C for 18 h. A single colony was then selected and transferred to MRS liquid medium. The culture was incubated at 37 °C with shaking at 200 *rpm* until the optical density at 600 nm reached 0.6, indicating the logarithmic growth phase. Subsequently, the culture was inoculated at a 1:100 ratio into fresh MRS liquid medium and incubated at 37 °C with shaking at 200 *rpm*. The supernatant of *W. confusa* RY-1 was collected every hour by centrifugation, and its pH value was measured.

### 2.2 Determination of antimicrobial activity

The antimicrobial activity of *W. confusa* RY-1 against pathogenic bacteria was evaluated using the agar diffusion method with Oxford cups. *W. confusa* RY-1 was cultured in MRS broth at 37 °C for 16–18 h, after which the supernatant was collected by centrifugation at 9000 *g* for 10 min at 4 °C and sterilized by filtration through a 0.22 μm membrane. Pathogenic strains—including carbapenem-resistant *Escherichia coli*, *Salmonella typhimurium*, and *Shigella flexneri*—were adjusted to a 0.5 McFarland standard using sterile PBS and uniformly spread onto LB agar plates. After drying, Oxford cups were placed on the agar, and 100 μL of the *W. confusa* RY-1 supernatant was added to each cup. The plates were first kept at 4 °C for 4 h to allow diffusion, and then incubated at 37 °C for 24 h. The diameter of the inhibition zones was measured in millimeters.

### 2.3 Animals and colitis modeling

A total of 40 healthy 7-8-week-old C57BL/6 J mice were supplied by Beijing Biotechnology Co., Ltd. (Beijing, China). All procedures for animals involved in the experiment were approved by the Animal Ethics Committee of Zunyi Medical University (Approval No. zyfy-an-2024-0258). The mice were housed under specific-pathogen-free (SPF) conditions with a 12-h light/dark cycle and provided with free access to food and water. They were acclimatized for 1 week prior to the experiment. A colitis model was induced by administering 1.5% DSS (molecular weight 36–50 kDa; MP Biomedicals, Santa Ana, USA) in the drinking water. The experimental grouping and treatment protocols were as follows: (1) Control group (n = 8): Mice received regular drinking water and were orally administered 200 μL of PBS daily for 10 days; (2) DSS group (n = 12): Mice received 1.5% DSS solution and were orally administered 200 μL of PBS daily for 10 days; (3) DSS + RY-1 strain group (n = 12): Mice received 1.5% DSS solution and were orally administered 200 μL of *W. confusa* RY-1 suspension (1 × 10^9^ CFU/mL) daily for 10 days, equivalent to 2 × 10^8^ CFU viable bacteria per mouse per day; (4) The RY-1 strain alone group (n = 8): Mice received regular drinking water and were orally administered 200 μL of *W. confusa* RY-1 suspension (1 × 10^9^ CFU/mL) daily for 10 days, equivalent to 2 × 10^8^ CFU viable bacteria per mouse per day. Body weight was recorded daily throughout the experimental period. Throughout the entire formal modeling period, only one mouse in the DSS + RY-1 strain group died accidentally due to an operational error, while all other experimental mice survived to the end of the study. On the evening of the 10th day of the experiment, the mice were fasted for 12 h (with access only to water). On the morning of day 11, the mice were euthanized: they were anesthetized with 2% isoflurane. After confirming that the pain had completely disappeared through toe pinch and corneal reflex detection and reaching a state of deep anesthesia, euthanasia by cervical dislocation was carried out. Immediately after euthanasia, mice were dissected. The colon was photographed and its length was measured, followed by collection of colonic contents, cecal contents and colonic tissue samples. Parts of the colonic tissues was fixed for histological analysis, while the remaining specimens were snap-frozen in liquid nitrogen and stored at −80 °C for subsequent assays.

### 2.4 Disease activity index assessment

The Disease Activity Index (DAI) was calculated daily from day 1 to day 10 based on three parameters: weight loss, stool consistency, and fecal occult blood (Novak et al., 2023). Each parameter was scored from 0 to 4, and the sum of these scores represented the final DAI value, reflecting the severity of DSS-induced colitis in mice. Fecal occult blood was detected using a commercial kit (Nanjing Jiancheng Bioengineering Institute, China).

### 2.5 Histopathological analysis

Upon euthanasia, tissue samples were randomly collected with six mice each from the control and the RY-1 strain alone groups, and nine mice each from the DSS and DSS + RY-1 strain groups. Segments of distal colon tissue (1–2 cm from the anus) were dissected, fixed in neutral buffered formalin for 24 h, and subsequently embedded in paraffin. Sections with a thickness of 4 μm were prepared and stained with hematoxylin and eosin (H&E). Histopathological damage was evaluated in a blinded manner using established criteria (Jang et al., 2019), including epithelial loss, crypt damage, goblet cell depletion, and inflammatory cell infiltration. For goblet cell counting, six tissue samples were collected from each of the control and RY-1 strain groups, and seven tissue samples were obtained from each of the DSS and DSS + RY-1 strain groups for paraffin section preparation. Following deparaffinization, the sections were stained using an Alcian blue staining kit (Leagene, Beijing, China), then observed and imaged under a microscope. Goblet cells were counted using FIJI (ImageJ) software (Wu et al., 2020; Chen Z. et al., 2024).

### 2.6 Immunofluorescence analysis

For immunofluorescence staining, five paraffin-embedded sections were randomly selected from each group. After deparaffinization and antigen retrieval, the sections were blocked with 5% goat serum at room temperature for 1 h, followed by incubation with primary antibodies against MUC2 (27675-1-AP, 1:400, Proteintech, Wuhan, China), ZO-1 (Ab221547, 1:100, Abcam, Shanghai, China), and Occludin (27260-1-AP, 1:500, Proteintech, Wuhan, China) at 4 °C overnight. After three washes with PBS, sections were incubated with corresponding secondary antibodies (SA00013-2, 1:300, Proteintech, Wuhan, China) for 1 h at room temperature and counterstained with DAPI to visualize nuclei. Images were acquired at 200x magnification using a Leica fluorescence microscope (Leica, Germany), with camera exposure time, excitation light intensity, and signal gain kept constant across all samples to avoid signal variability. Fluorescence intensity was quantified with FIJI (ImageJ) software. The collected single-channel fluorescence images were imported into the FIJI (ImageJ) software and converted into grayscale images. A uniform grayscale threshold was used to define the fluorescence positive areas for all sections. The pixel area and mean fluorescence intensity (Mean) of the positive region were detected in each section. The mean fluorescence intensity (Mean) of the positive region was used as the quantitative indicator of the fluorescence signal to directly characterize the relative expression level of the target protein.mean fluorescence intensity.

### 2.7 16S rRNA sequencing analysis

Upon dissection, colonic contents were aseptically collected from all mice, immediately frozen in liquid nitrogen, and stored at −80 °C. Subsequently, genomic DNA was extracted from colonic content samples, and the V3–V4 hypervariable region of the bacterial 16S rRNA gene was amplified with primers 341F and 805R (Logue et al., 2016). Amplified products were purified with AMPure XT beads, and library quality was assessed using an Agilent 2,100 Bioanalyzer (Agilent, USA) and the Illumina Library Quantification Kit (Kapa Biosciences, USA). Libraries with concentrations exceeding 2 nM were subjected to paired-end sequencing (2 × 250 bp) on an Illumina NovaSeq 6,000 platform. Raw sequencing data were processed and analyzed using the QIIME2 pipeline.

### 2.8 Untargeted metabolomic analysis by GC–MS

Upon dissection, cecal contents were randomly collected from six mice per group, immediately frozen in liquid nitrogen, and stored at −80 °C. Subsequently, 50 mg of the cecal contents was weighed and transferred to a 2 mL grinding tube, followed by the addition of 500 μL of aqueous methanol solution and homogenization at −20 °C. After ultrasonic extraction, the sample was centrifuged at 4 °C and 13,000 *g* for 15 min. The supernatant was transferred to a glass derivatization vial and dried under a gentle stream of nitrogen. Subsequently, 80 μL of methoxyamine hydrochloride in pyridine was added, and the mixture was incubated at 37 °C with shaking for 90 min. Then, 80 μL of N, O-bis(trimethylsilyl)trifluoroacetamide with 1% trimethylchlorosilane (BSTFA + 1% TMCS) was added as a derivatization reagent, and the reaction continued at 70 °C for 60 min. After cooling to room temperature, the mixture was allowed to stand for 30 min, and then the sample was injected into the GC–MS system for analysis.

### 2.9 Transcriptome sequencing and analysis

Colon tissue samples were randomly selected from three mice in each group, flash-frozen in liquid nitrogen, and stored at −80 °C. Subsequent steps including RNA purification, reverse transcription, library construction, and sequencing were conducted by Shanghai Majorbio Bio-pharm Biotechnology Co., Ltd. (Shanghai, China) following the manufacturer’s protocols. Total RNA was extracted from colon tissues of the control, DSS, DSS + RY-1 strain, and the RY-1 strain alone groups using the Qiagen RNA Extraction and Purification Kit. RNA integrity was evaluated by agarose gel electrophoresis, and purity was measured with a NanoDrop2000 spectrophotometer (Thermo Fisher Scientific, USA). RNA concentration was accurately determined using a Qubit 4.0 Fluorometer (Thermo Fisher, USA), and integrity was further verified on an Agilent 5,300 Fragment Analyzer (Agilent, USA). After passing quality control, libraries were prepared with the Illumina® Stranded mRNA Prep Kit (San Diego, CA, USA). Qualified libraries from different groups were pooled based on effective concentration and sequencing depth requirements, followed by sequencing on an Illumina platform to generate 150 bp paired-end reads. Bioinformatics analysis included quality control, sequence alignment, transcript assembly, gene expression quantification, differential expression analysis, and functional enrichment analysis of differentially expressed genes. The parameters of |logFC| > 2 and *P*-adj < 0.05 (*P*-adj represents the corrected *p*-value) were used as the screening criteria for differential genes.

### 2.10 Statistical analysis

All data are expressed as the mean ± standard error of the mean (SEM) and were analyzed using SPSS software (version 29.0). Comparisons between two groups were made using the independent samples t-test. For comparisons involving three or more groups, homogeneity of variances was first assessed using Levene’s test. As the assumption of equal variances was violated among some groups (*p* < 0.05), Welch’s ANOVA (which does not assume homogeneity of variances) was employed, followed by Dunnett’s T3 post-hoc test for multiple comparisons. A *p*-value of less than 0.05 was considered statistically significant.

## 3 Results

### 3.1 Acid production and antibacterial activity of *W. confusa* RY-1

**Figure 1:**
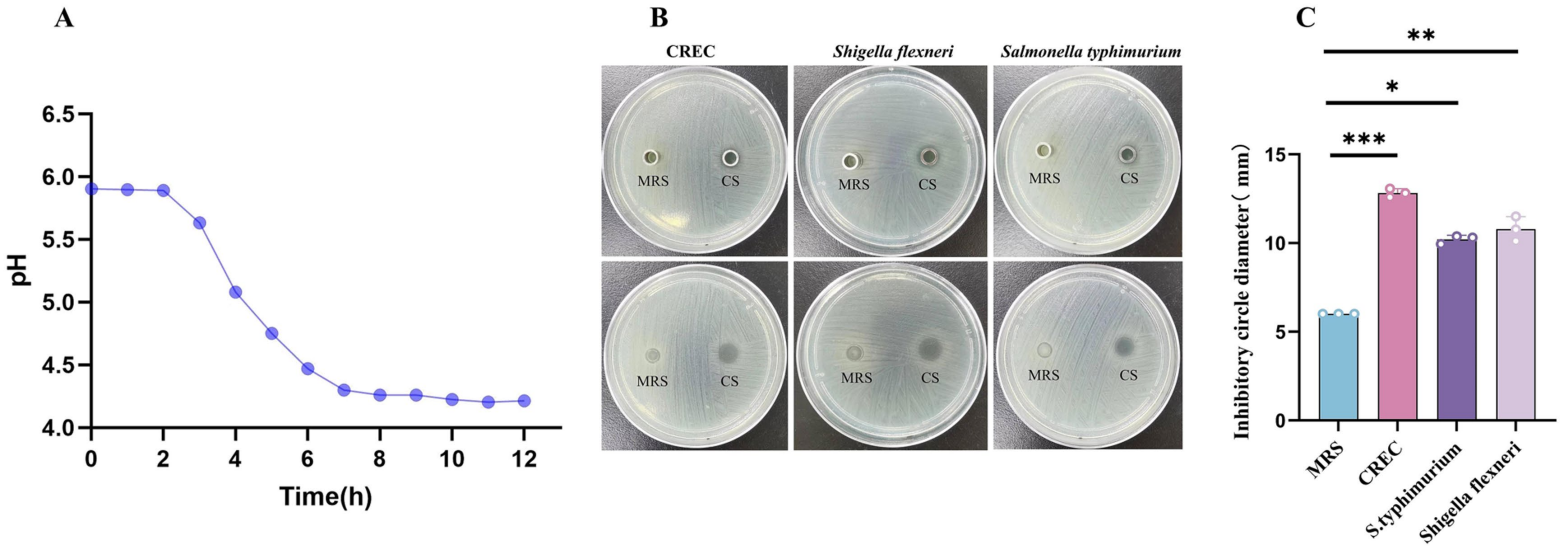
Acid production and antibacterial activity of *W. confusa* RY-1 **(A)** pH changes during the growth of *W. confusa* RY-1 **(B)** Inhibitory activity of *W. confusa* RY-1 against pathogenic bacteria **(C)** Diameters of inhibition zones. Data are presented as mean ± SEM, *n* = 3, *p < 0.05, **p < 0.01, ***p < 0.001.

Previous studies have indicated that *W. confusa* is capable of producing organic acids. Therefore, we assessed the acid-producing capacity of *W. confusa* RY-1. The results demonstrated that the pH of the culture medium decreased gradually with prolonged incubation time and reached a plateau after 7 h (Figure 1A), indicating a pronounced acid-producing ability of *W. confusa* RY-1. Furthermore, we evaluated the antibacterial activity of *W. confusa* RY-1 using an agar well diffusion assay. The results revealed that, compared to the MRS control group, *W. confusa* RY-1 exhiba well diffusion assayited significant antibacterial effects against carbapenem-resistant *Escherichia coli*, *Salmonella typhimurium*, and *Shigella flexneri* (Figures 1B,C). These results suggest that *W. confusa* RY-1 possesses probiotic potential.

### 3.2 *W. confusa* RY-1 attenuates DSS-induced colitis in mice

**Figure 2:**
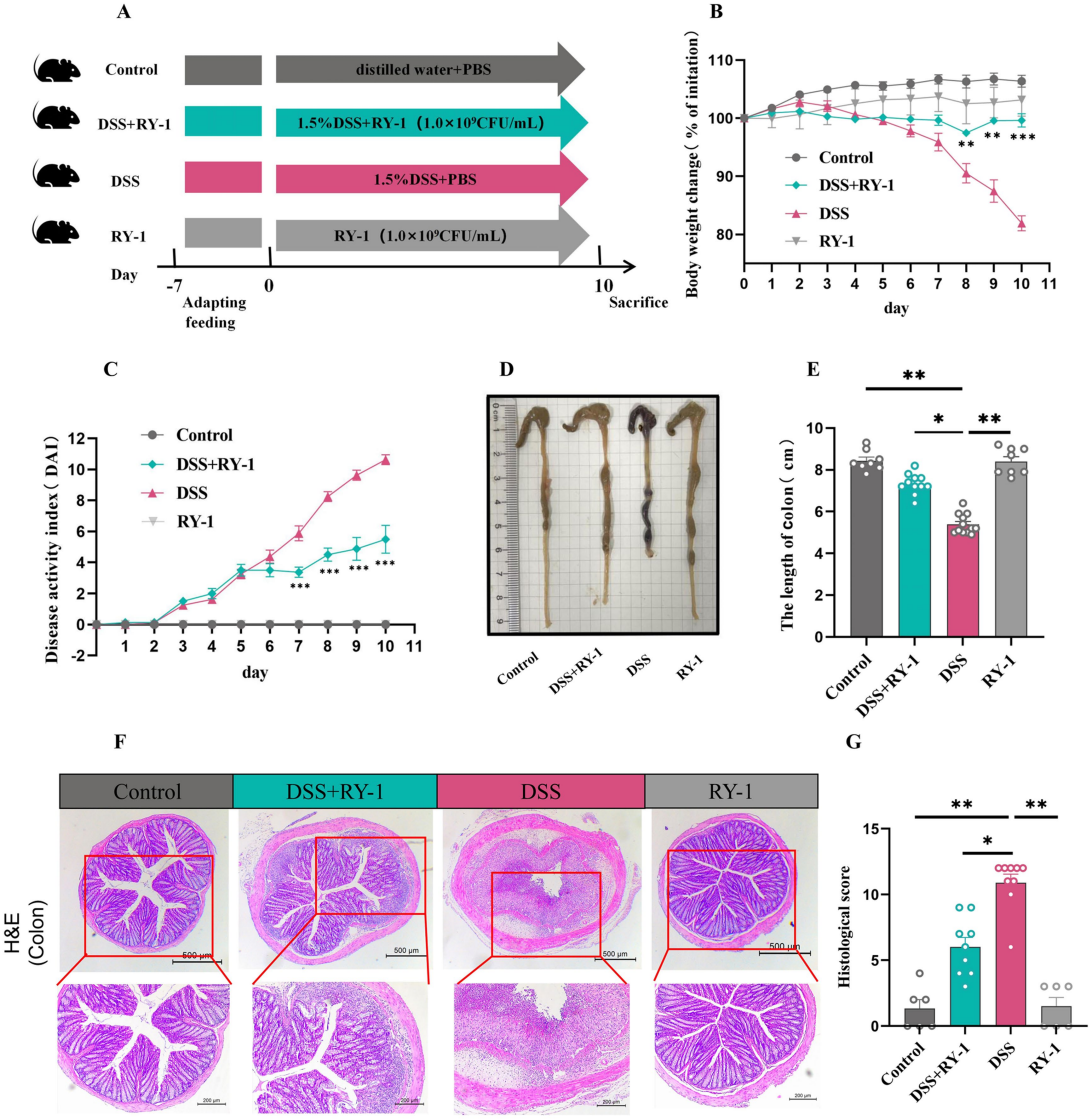
Ameliorative effects of *W. confusa* RY-1 on DSS-induced colitis symptoms. **(A)** Experimental design schematic. **(B)** Daily changes in body weight. **(C)** Daily DAI scores. **(D)** Representative images of colons from each group. **(E)** Quantification of colon length (*n* = 8–12). **(F)** Representative images of H&E-stained colon tissues (scale bar = 500 μm) with corresponding magnified views (scale bar = 200 μm). **(G)** Histological scores of colon sections (*n* = 6–9). Data are presented as mean ± SEM; *p < 0.05, **p < 0.01, ***p < 0.001. “RY-1” in the figure panels and legend denotes the “RY-1 strain” (i.e., *W. confusa* RY-1).

Next, we investigated whether *W. confusa* RY-1 exerts a protective effect against DSS-induced colitis in mice. Experimental colitis was induced by administering 1.5% DSS in drinking water continuously for 10 days. Concurrently, *W. confusa* RY-1 was administered daily via oral gavage to evaluate its therapeutic potential (Figure 2A). The results showed that compared to the control group, mice in the DSS group exhibited significant body weight loss, increased disease activity index (DAI), and marked colon shortening. Notably, treatment with *W. confusa* RY-1 significantly ameliorated DSS-induced weight loss, reduced DAI scores, and attenuated colon shortening (Figures 2B–E). Furthermore, histopathological analysis revealed that the DSS group displayed disrupted mucosal architecture, crypt damage, and extensive inflammatory cell infiltration in colonic tissues compared to the control group. In contrast, *W. confusa* RY-1 treatment helped preserve the intestinal mucosal structure, mitigated the loss of goblet cells, and reduced inflammatory cell infiltration. The histological scores of the treated group were significantly lower than those of the DSS group (Figures 2F–G). Collectively, these results indicate that *W. confusa* RY-1 effectively attenuates DSS-induced colitis in mice. In addition, the RY-1 strain alone group without DSS treatment was included in this study to evaluate the intestinal safety of the RY-1 strain in healthy mice. Statistical analysis of all parameters across groups showed that the RY-1 strain alone group and the normal control group exhibited no significant differences with respect to body weight changes, DAI scores, colon length, or histopathological injury scores. These results suggest that administration of the RY-1 strain alone does not cause colonic damage in mice, indicating that the RY-1 strain has no inherent intestinal toxicity. This further supports that the ameliorative effect of the RY-1 strain on DSS-induced colitis is attributable to its specific anti-inflammatory action.

### 3.3 *W. confusa* RY-1 alleviates DSS-induced intestinal barrier impairment

**Figure 3:**
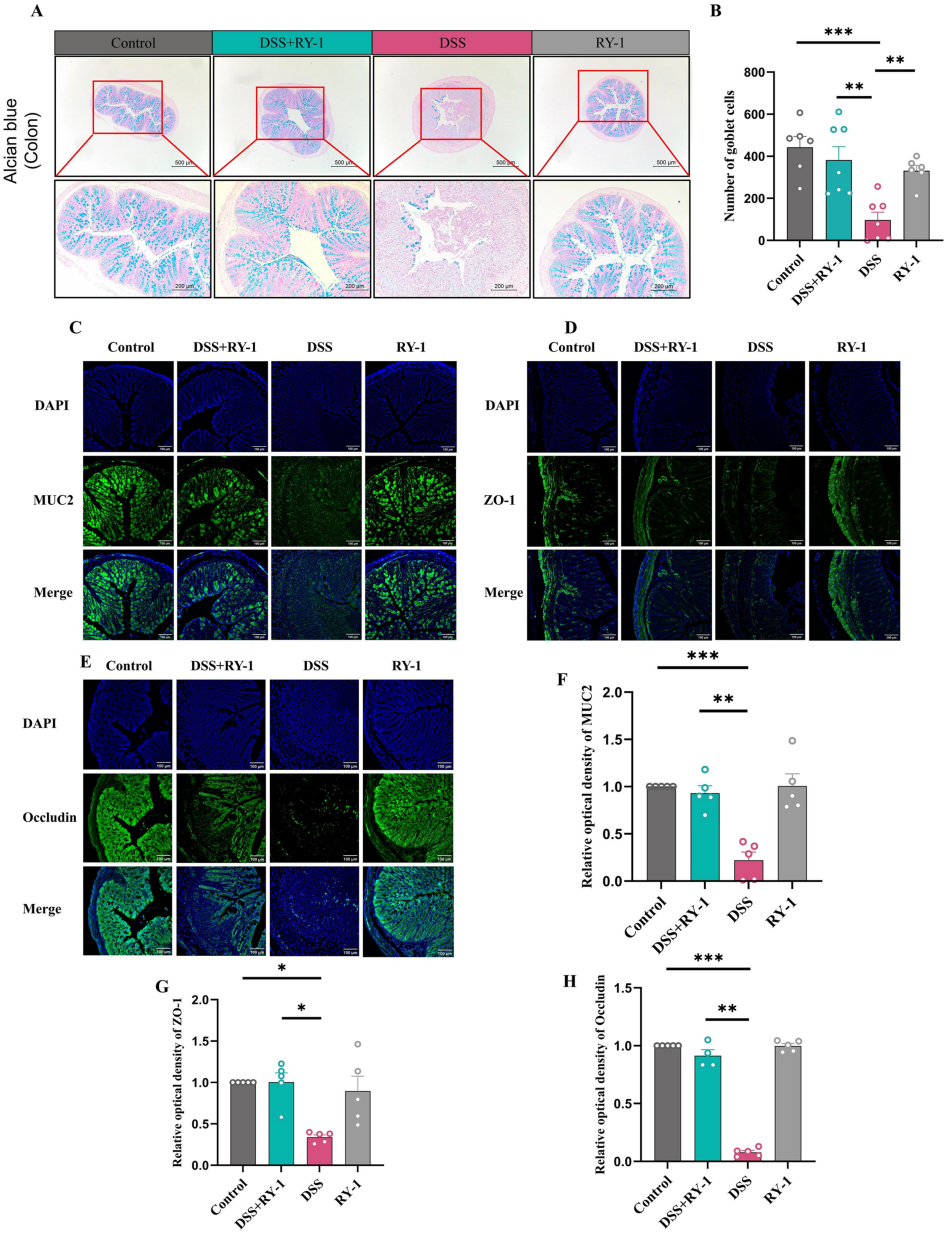
*W. confusa* RY-1 alleviates DSS-induced intestinal barrier damage. **(A)** Representative images of Alcian Blue-stained colon sections (scale bar = 500 μm) with corresponding magnified views (scale bar = 200 μm). **(B)** Quantification of goblet cell numbers (*n* = 6–7). **(C)** Representative immunofluorescence images of MUC2 in colon tissues (scale bar = 100 μm). **(D)** Representative immunofluorescence images of ZO-1 (scale bar = 100 μm). **(E)** Representative immunofluorescence images of Occludin (scale bar = 100 μm). **(F)** Relative optical density of MUC2. **(G)** Relative optical density of ZO-1. **(H)** Relative optical density of Occludin (*n* = 4–5). Data are presented as mean ± SEM; *p < 0.05, **p < 0.01, ***p < 0.001. “RY-1” in the figure panels and legend denotes the “RY-1 strain” (i.e., *W. confusa* RY-1).

Since impairment of the mucus layer secreted by goblet cells compromises intestinal barrier integrity and increases the risk of colitis (Stolfi et al., 2022; Yu et al., 2024), we evaluated whether *W. confusa* RY-1 influences mucus production. Alcian blue staining of goblet cells and immunofluorescence staining for mucin-2 (MUC2) were performed on mouse colonic tissues. The results revealed that both goblet cell numbers and MUC2 expression were significantly reduced in DSS-treated mice compared with the control group. Treatment with *W. confusa* RY-1 significantly attenuated the DSS-induced loss of goblet cells and MUC2 expression (Figures 3A–C,F). Furthermore, given the critical role of tight junction proteins inmaintaining intestinal barrier integrity, we examined the effect of *W. confusa* RY-1 on their expression. Immunofluorescence staining for ZO-1 and Occludin was carried out using specific antibodies. The results indicated that expression levels of both ZO-1 and Occludin were significantly decreased in the DSS group relative to controls. Notably, *W. confusa* RY-1 administration markedly restored the expression of these tight junction proteins (Figure 3D–E,G,H). Together, these findings demonstrate that *W. confusa* RY-1 helps protect against DSS-induced damage to the intestinal barrier.

### 3.4 *W. confusa* RY-1 modulates the composition of the gut microbiota

**Figure 4:**
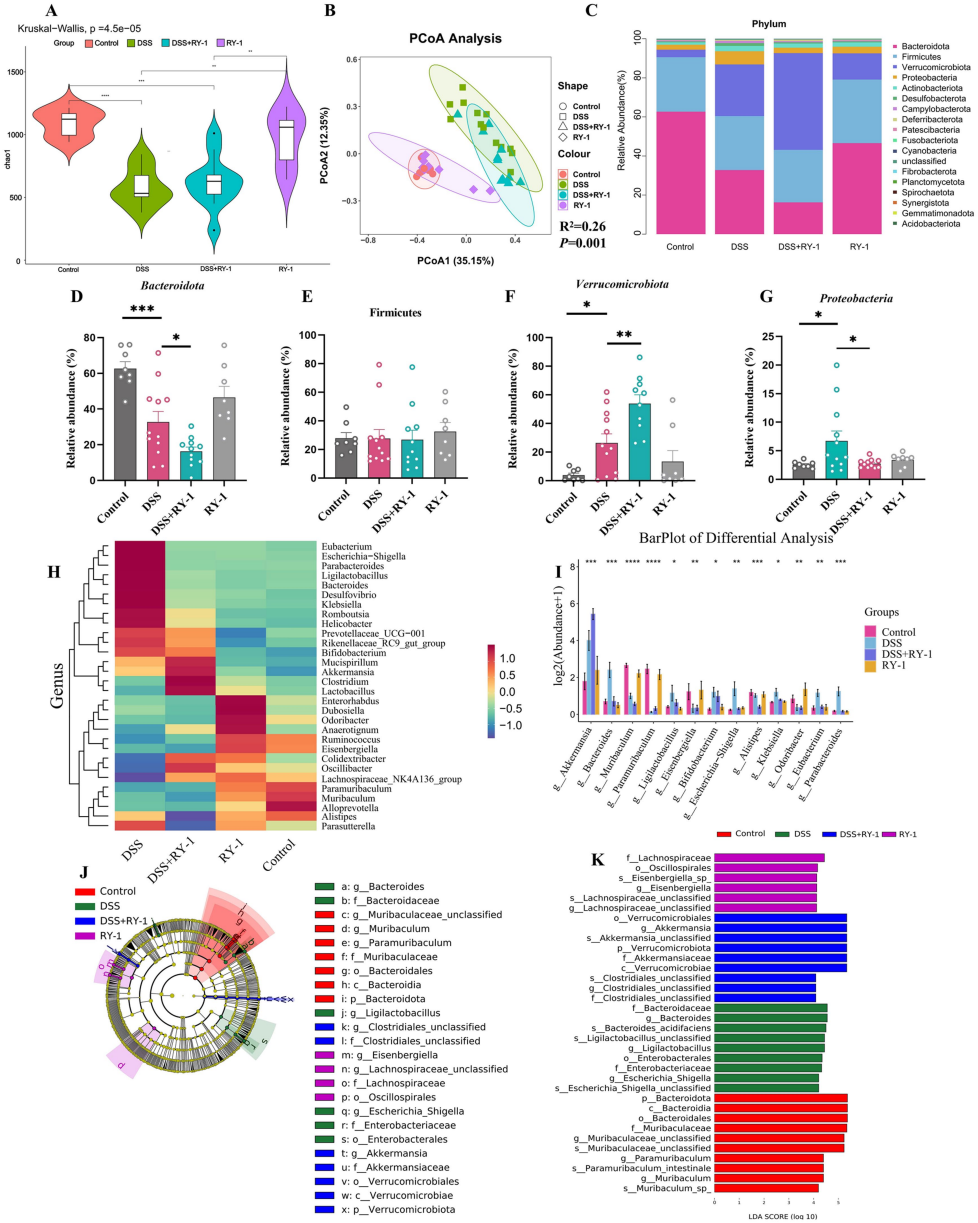
Modulation of gut microbiota in colon contents by *W. confusa* RY-1 treatment. **(A)** Alpha diversity assessed by the Chao1 index. **(B)** Principal coordinates analysis (PCoA) based on Bray–Curtis distances. **(C)** Relative abundance of bacterial communities at the phylum level. Relative abundance of key phyla: **(D)** *Bacteroidota*, **(E)** *Firmicutes*, **(F)** *Verrucomicrobiota*, and **(G)** *Proteobacteria* across the four groups. **(H)** Heatmap of relative abundance at the genus level. **(I)** Intergroup significance analysis of genus-level taxa. **(J)** LEfSe analysis indicating differentially abundant taxa. **(K)** Linear discriminant analysis (LDA) with a threshold score > 4.0. Data are presented as mean ± SEM; *n* = 8–12 per group; *p < 0.05, **p < 0.01, ***p < 0.001. “RY-1” in the figure panels and legend denotes the “RY-1 strain” (i.e., *W. confusa* RY-1).

The gut microbiota plays a critical role in the development of UC (Wang J. et al., 2020). To investigate whether *W. confusa* RY-1 influences the gut microbiota in mice with DSS-induced colitis, we performed 16S rRNA gene sequencing. Subsequent *α*-diversity analysis based on the Chao1 and Shannon indices further revealed that the RY-1 strain supplementation partially restored the richness (Chao1, Figure 4A) of the gut microbiota, but did not significantly affect the overall diversity (Shannon, Supplementary Figure S1). Although the Shannon index showed an increasing trend in the DSS + RY-1 group compared with the DSS group, the difference was not statistically significant. Principal coordinates analysis (PCoA) based on Bray–Curtis distances revealed a clear separation between the DSS and control groups. Notably, *W. confusa* RY-1 treatment also significantly shifted the microbial community structure (*R*^2^ = 0.26, *p* = 0.001; Figure 4B), indicating a substantial modulatory effect of *W. confusa* RY-1 on the gut microbiota in mice with colitis. At the phylum level, the dominant taxa in the fecal microbiota included *Bacteroidota*, *Firmicutes*, *Verrucomicrobiota*, and *Proteobacteria* (Figure 4C). Compared with the control group, the DSS group exhibited a significant increase in the relative abundances of *Verrucomicrobiota* and *Proteobacteria*, a decrease in *Bacteroidota*, and no significant change in *Firmicutes*. Treatment with *W. confusa* RY-1 significantly reduced the relative abundances of *Bacteroidota* and *Proteobacteria*, increased *Verrucomicrobiota*, but did not significantly alter *Firmicutes* compared to the DSS group. To further examine changes in bacterial abundance (Figures 4D–G), we performed hierarchical clustering of the top 30 genera and generated a heatmap for detailed analysis (Figure 4H). The DSS group showed a significant increase in the relative abundance of *Escherichia–Shigella*, *Parabacteroides*, and *Klebsiella* compared to the control group. These changes were effectively reversed by *W. confusa* RY-1 treatment (Figures 4H–I). We further conducted LEfSe analysis with an LDA score threshold of 4.0 to identify differentially enriched taxa between the DSS and DSS + RY-1 groups (Figures 4J–K). The results indicated significant enrichment of potentially pathogenic bacteria, including *Enterobacterales*, *Enterobacteriaceae*, *Escherichia–Shigella*, and *Klebsiella*, in the DSS group. In contrast, the DSS + RY-1 group showed increased abundance of beneficial bacteria such as *Akkermansiaceae* and *Akkermansia*. Collectively, these results indicate that *W. confusa* RY-1 significantly restructures the gut microbiota in mice with colitis.

### 3.5 *W. confusa* RY-1 alters the gut metabolome in colitis mice

**Figure 5:**
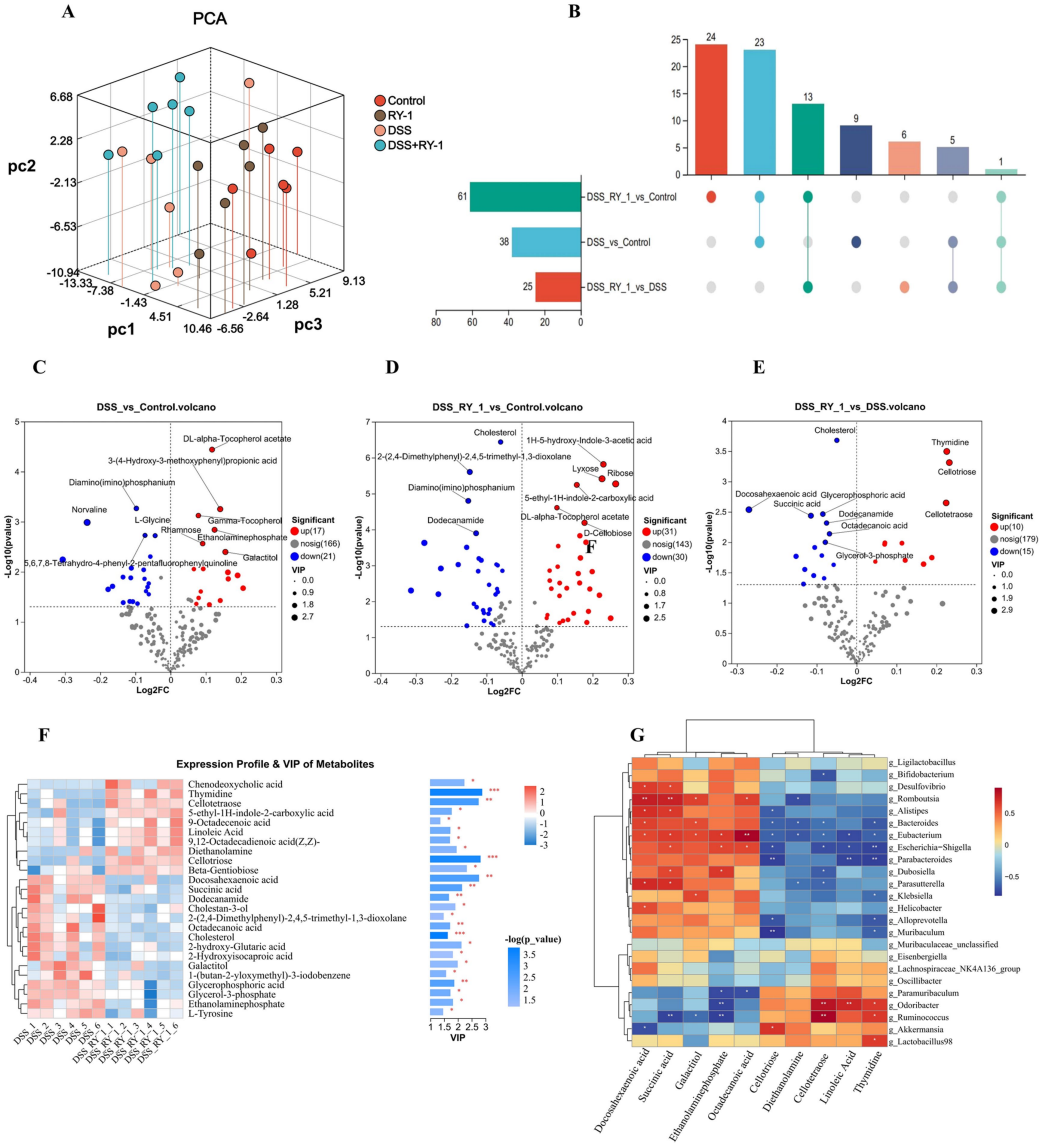
Effects of *W. confusa* RY-1 on intestinal metabolism in mice. **(A)** PCA score plot of metabolome profiles from the four groups. **(B)** Number of differential metabolites identified in each comparison. **(C)** Volcano plot of metabolites: DSS vs. control. **(D)** Volcano plot of metabolites: DSS + RY-1 strain vs. control. **(E)** Volcano plot of metabolites: DSS + RY-1 strain vs. DSS. **(F)** Clustered heatmap and variable importance in projection (VIP) scores of differential metabolites between DSS + RY-1 strain and DSS groups. **(G)** Correlation analysis between gut microbiota and metabolites. Data are presented as mean ± SEM (*n* = 6), *p < 0.05, **p < 0.01, ***p < 0.001. “RY-1” in the figure panels and legend denotes the “RY-1 strain” (i.e., *W. confusa* RY-1).

The gut microbiota influences intestinal homeostasis through its metabolites, which act as key mediators between microbial communities and the host (Lavelle and Sokol, 2020b). To examine the effect of *W. confusa* RY-1 on the metabolomic profile in mice with DSS-induced colitis, we performed untargeted metabolomics analysis on samples of cecal contents from each group. The three-dimensional PCA score plot illustrates the global metabolic variation among the four experimental groups. A clear separation between the control and DSS groups suggested that DSS challenge induced profound metabolic reshaping. The RY-1 strain alone group clustered closely with the control group, indicating that administration of the RY-1 strain alone had negligible effects on the basal metabolic profile. Notably, the DSS + RY-1 strain group occupied an intermediate position between the DSS and control groups, suggesting that treatment with the RY-1 strain partially attenuated DSS-induced metabolic dysregulation and shifted the metabolic profile closer to that of the control group (Figure 5A). Venn analysis revealed 61, 38, and 25 differential metabolites in the comparisons DSS + RY-1 strain vs. control, DSS vs. control, and DSS + RY-1 strain vs. DSS, respectively. Additionally, 24, 9, and 6 metabolites unique to each comparison were identified in these three comparison sets (Figure 5B). Differential metabolite analysis revealed 17 upregulated and 21 downregulated metabolites in the DSS vs. control comparison (Figure 5C); 31 upregulated and 30 downregulated in the DSS + RY-1 strain vs. control comparison (Figure 5D); and 10 upregulated and 15 downregulated metabolites in the DSS + RY-1 strain vs. DSS comparison (Figure 5E). Compared with the DSS group, the DSS + RY-1 strain group exhibited increased levels of cellotriose, linoleic acid, and cellotetraose, and decreased levels of succinic acid, octadecanoic acid, and glycerophosphoric acid (Figure 5F). Spearman correlation analysis indicated that succinic acid was positively correlated with *Escherichia–Shigella*, while linoleic acid showed a positive correlation with cellotriose and cellotetraose, and a negative correlation with *Escherichia–Shigella* (Figure 5G). These results suggest that *W. confusa* RY-1 remodels the gut metabolic landscape in mice with colitis.

### 3.6 Transcriptomic analysis of colon tissue from colitis mice treated with *W. confusa* RY-1

**Figure 6:**
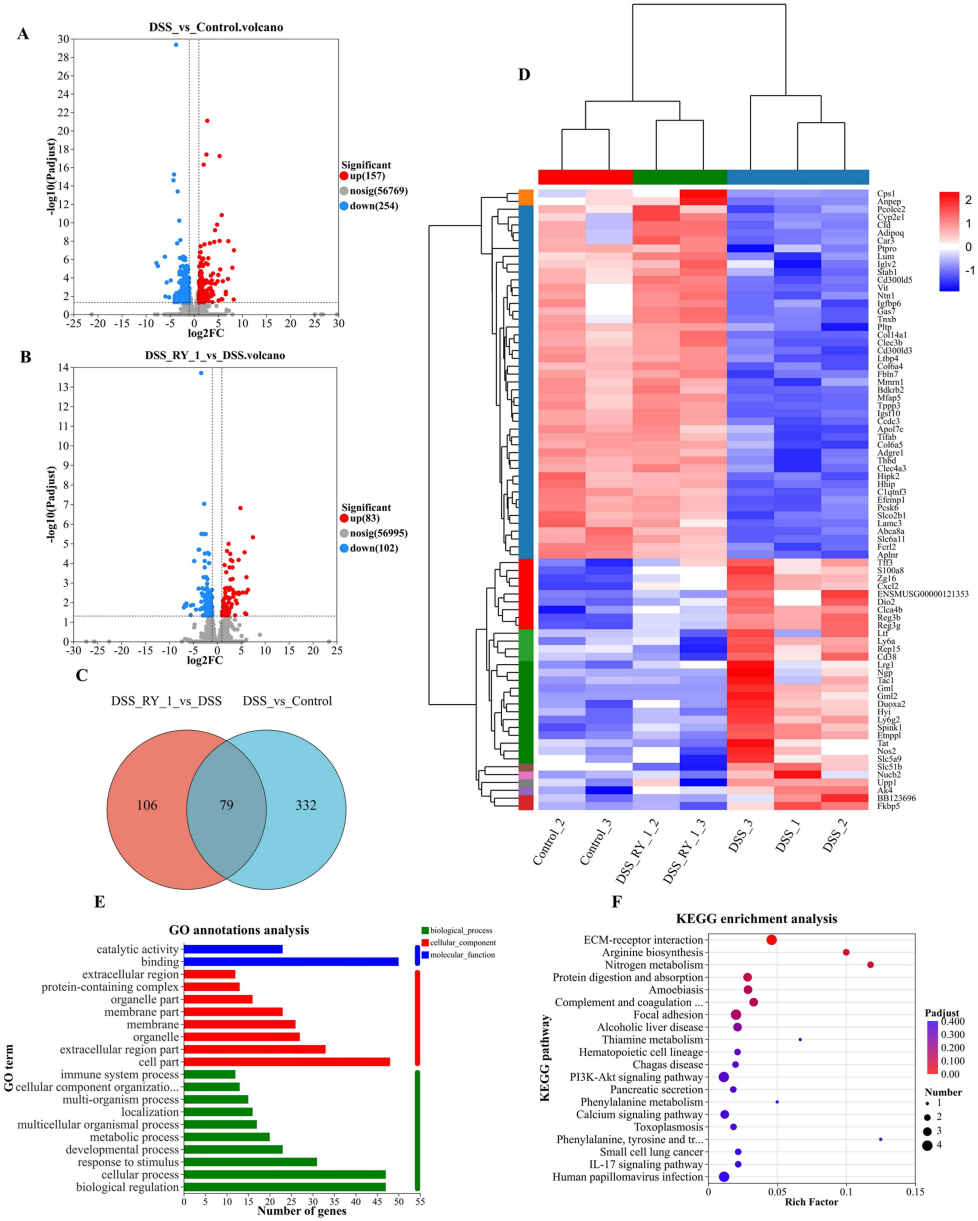
Transcriptomic analysis of colon tissues from colitis mice treated with *W. confusa* RY-1. **(A)** Volcano plot of differentially expressed genes (DEGs) between the control and DSS groups. **(B)** Volcano plot of DEGs between the DSS and DSS + RY-1 strain groups. **(C)** Venn diagram illustrating overlapping DEGs among the control, DSS, and DSS + RY-1 strain groups. **(D)** Hierarchical clustering heatmap of DEGs across groups. **(E)** GO enrichment analysis of DEGs regulated by *W. confusa* RY-1. **(F)** KEGG pathway enrichment analysis of DEGs regulated by *W. confusa* RY-1 (*n* = 3). “RY-1” in the figure panels and legend denotes the “RY-1 strain” (i.e., *W. confusa* RY-1).

To further elucidate the mechanism by which *W. confusa* RY-1 ameliorates DSS-induced colitis, we performed transcriptome sequencing on colon tissues from each group. Compared to the control group, the DSS group exhibited 157 upregulated and 254 downregulated genes (Figure 6A). In contrast, treatment with *W. confusa* RY-1 resulted in 83 upregulated and 102 downregulated genes relative to the DSS group (Figure 6B). A Venn diagram showed that 79 differentially expressed genes in the DSS group were reversed by *W. confusa* RY-1 treatment (Figure 6C). Cluster analysis heatmap revealed significant differences in gene expression levels between the DSS group and the DSS + RY-1 strain group. Among them, the expression levels of S100a8, a key pro-inflammatory alarmin gene (Jakobsson et al., 2023), and Zg16, a gene related to the composition of mucus (Ma et al., 2023), were significantly restored by *W. confusa* RY-1 (Figure 6D). Gene Ontology (GO) analysis indicated that the genes modulated by *W. confusa* RY-1 were primarily involved in biological regulation, cellular processes, response to stimulus, and metabolic functions (Figure 6E). KEGG pathway analysis further demonstrated that the differentially expressed genes were mainly enriched in the ECM–receptor interaction pathway (Figure 6F). Collectively, these transcriptomic findings suggest that *W. confusa* RY-1 alleviates DSS-induced colitis by restoring gene expression patterns related to inflammation, mucosal integrity, and metabolic processes.

## 4 Discussion

Acid production is a key characteristic of probiotics, which enables them to generate substantial amounts of organic acids that reduce environmental pH and inhibit the growth of pathogenic bacteria (Zhang et al., 2024). Previous studies have reported that *Weissella* strains can produce bacteriocins, hydrogen peroxide, and organic acids, which suppress foodborne pathogens (Teixeira et al., 2021). In this study, *W. confusa* RY-1 exhibited strong acid-producing capacity and significantly inhibited the growth of three intestinal pathogenic bacteria through acid production, highlighting its potential probiotic value. This probiotic potential is further supported by the fact that *Weissella* strains are frequently isolated from the human gastrointestinal tract (Wang W. et al., 2020). Prior research has shown that *W. confusa* can alleviate inflammatory responses, obesity, and constipation, reduce hypercholesterolemia, and display broad-spectrum antimicrobial activity (Goh and Philip, 2015; Ahmed et al., 2022; Sun et al., 2024; Javed et al., 2025; Ki et al., 2025). Our findings further demonstrate that *W. confusa* RY-1, isolated from human gastric fluid, confers protection against DSS-induced colitis in mice, as evidenced by the attenuation of body weight loss, reduction of DAI scores, and prevention of colon shortening.

Goblet cells are essential for maintaining the structural integrity of the intestinal epithelium and serve as the main source of the mucin MUC2 (Wang Q. et al., 2022), which is the major constituent of colonic mucus. Previous studies have demonstrated that patients with UC exhibit a reduction in goblet cell numbers, decreased MUC2 expression, and thinning of the mucus layer (Johansson et al., 2014; van der Post et al., 2019). Consistent with these observations, DSS-induced colitis in mice led to goblet cell loss and a marked downregulation of MUC2. Notably, treatment with *W. confusa* RY-1 significantly counteracted these pathological changes by elevating MUC2 levels and restoring goblet cell populations. This suggests that *W. confusa* RY-1 contributes to the maintenance of intestinal epithelial integrity, aligning with previous reports on other probiotics (Wang W. et al., 2020; Wu et al., 2022). In addition, the tight junction proteins ZO-1 and Occludin play a vital role in preserving the intestinal mucosal barrier (Pan et al., 2020), and their dysregulation is closely linked to UC pathogenesis (Al-Sadi et al., 2014). Interestingly, *W. confusa* RY-1 also markedly upregulated the expression of ZO-1 and Occludin, further confirming its efficacy in promoting intestinal barrier repair. Together, these findings indicate that *W. confusa* RY-1 ameliorates colitis, an effect associated with the restoration of goblet cell function and enhancement of tight junction protein expression, thereby strengthening the intestinal mucosal barrier.

Accumulating evidence indicates that the gut microbiota is closely linked to the pathogenesis and treatment of UC (He et al., 2019; Quraishi et al., 2019; Qi et al., 2022). Inflammation in UC not only reduces microbial diversity but also alters the composition of specific bacterial communities (Ni et al., 2017; Franzosa et al., 2019; Khan et al., 2019; Tian et al., 2020). Changes in the abundance of *Proteobacteria* are considered a microbial dysbiosis marker (Shin et al., 2015; Zhu et al., 2018). Consistent with previous studies (Shi et al., 2020; Wang J. et al., 2020), DSS treatment increased *Proteobacteria* abundance. We also found significantly elevated levels of *Escherichia–Shigella*, *Parabacteroides*, and *Klebsiella* in UC mice, all of which have been associated with intestinal barrier disruption and disease progression (Dogan et al., 2014; Peng et al., 2019; Chiang et al., 2021; Niu et al., 2022). Previous reports indicate that colitis development leads to expansion of *Escherichia–Shigella*, further disrupting microbial balance (Jialing et al., 2020). Our results confirmed that DSS induction markedly increased the abundance of *Escherichia–Shigella*; however, *W. confusa* RY-1 intervention restored its abundance to levels comparable to the control group. Collectively, these findings indicate that *W. confusa* RY-1 partially reversed DSS-induced gut microbiota dysbiosis at the community composition level, as evidenced by the recovery of species richness (Chao1) and the reduction of pathobionts such as *Escherichia-Shigella*. Nevertheless, the decrease in the Shannon index suggests that community evenness was not simultaneously restored, suggesting that RY-1 strain modulation primarily affected taxon-specific abundance rather than overall ecological balance.

Interactions between the gut microbiota and the host are largely mediated by microbial metabolites, which function as intermediates or end products of microbial metabolism (Lavelle and Sokol, 2020a). Growing evidence suggests that gut microbial metabolites contribute to the development of UC (Zou et al., 2020; Ji et al., 2023). In this study, *W. confusa* RY-1 significantly modulated fecal metabolite levels in UC mice, particularly linoleic acid (LA) and succinic acid. LA, an omega-6 polyunsaturated fatty acid, is often reduced in UC patients and mice with colitis (Belury, 2023; Jia et al., 2024). *W. confusa* RY-1 significantly increased LA levels in the feces of UC mice, suggesting that LA may contribute to colitis alleviation through anti-inflammatory effects and barrier repair. Additionally, succinic acid, an intermediate metabolite in short-chain fatty acid synthesis, is elevated in UC patients and experimental colitis (Ariake et al., 2000; Osaka et al., 2017; Fremder et al., 2021). Treatment with *W. confusa* RY-1 significantly reduced succinic acid levels, further supporting the anti-inflammatory effect of this reduction, as succinate accumulation has been linked to intestinal inflammation (Connors et al., 2018). However, this study has limitations, including the lack of direct functional validation for certain metabolic findings. Future research should further evaluate the specific effects of these metabolites in UC.

Transcriptome sequencing revealed increased expression of the *S100a8* gene in the DSS group. *S100a8*, a member of the S100 protein family, is mainly expressed in neutrophils and monocytes and has been implicated in various inflammatory diseases, including inflammatory bowel disease (IBD) (Ran et al., 2025). As a damage-associated molecular pattern, *S100a8* can disrupt intestinal mucosal homeostasis and accelerate colitis progression by activating pro-inflammatory signaling cascades (Shi et al., 2022). Notably, *W. confusa* RY-1 intervention restored *S100a8* expression to levels comparable to the normal control group, suggesting that its anti-colitis effect may involve suppression of *S100a8*-mediated inflammation, although the exact regulatory mechanisms require further investigation. Zymogen granule protein 16 (*Zg16*) is a secretory glycoprotein found in zymogen granules (Chen X. et al., 2024). It collaborates with the MUC2 network to help maintain a protective distance between bacteria and the epithelial surface (Bergstrom et al., 2016). Existing studies confirm that *Zg16* deficiency leads to impaired mucosal barrier function and microbial dysbiosis, triggering low-grade inflammation that may drive the onset and progression of ulcerative colitis and inflammation-associated colorectal cancer (Chen X. et al., 2024). In this study, *Zg16* expression was increased in the DSS group, possibly as a compensatory response to DSS-induced epithelial damage. Given the complexity of host–microbe transcriptional crosstalk, further studies are needed to clarify the functional role of microbiota in IBD pathogenesis.

Li et al. reported that *Lactobacillus acidophilus* 6,074 fermented jujube juice ameliorates colitis by protecting the intestinal barrier, regulating inflammation, and modulating the gut microbiota (Li et al., 2025)—pathways that are largely consistent with those identified in the present study. However, the two intervention systems are fundamentally distinct. Their study employed a co-fermentation system of jujube pulp and lactic acid bacteria, which relied on the synergistic effects of bioactive compounds from jujube, and only examined basic inflammatory cytokines and phylum-level microbiota changes, without conducting *in vitro* functional assays of the pure strain, safety evaluations, mucin-regulatory investigations, or multi-omics correlation analyses. In contrast, the present study adopted purified *W. confusa* RY-1 as a standalone intervention, excluding interference from plant-derived components, and demonstrated its acid-producing and antimicrobial properties in vitro, while confirming the absence of intestinal toxicity *in vivo*. Furthermore, we showed that the RY-1 strain partially restored the tight junctions and the mucus layer, and exerted anti-inflammatory effects at the transcriptional level via modulation of *S100a8*. By integrating 16S rRNA sequencing with cecal untargeted metabolomics, we further established microbiota-metabolite associations, addressing gaps in previous research. While the fermented jujube juice study provided a classical analytical framework for the present work, our study expands the theoretical basis for *Weissella*-based intervention in inflammatory bowel disease and offers a stand-alone probiotic strain resource.

This study still has certain limitations. No positive control group was established using first-line clinical colitis drugs such as 5-aminosalicylic acid or sulfasalazine; four groups were included: the normal control group, DSS model group, DSS + RY-1 strain group, and RY-1 strain alone group. Consequently, we were unable to directly and quantitatively compare the efficacy of the RY-1 strain with that of established anti-inflammatory drugs, which to some extent limits the comprehensive evaluation of RY-1’s clinical application potential. In future experiments, 5-aminosalicylic acid or sulfasalazine will be introduced as a positive control to quantitatively compare the therapeutic efficacy of RY-1 and further evaluate its therapeutic potential for colitis treatment.

## 5 Conclusion

*W. confusa* RY-1, a novel strain isolated from healthy human gastric fluid, was shown to ameliorate DSS-induced colitis by improving intestinal barrier function, modulating gut microbiota composition, regulating metabolic profiles, and influencing transcriptomic responses. This strain represents a promising probiotic candidate for the treatment of ulcerative colitis.

## Data Availability

The sequencing data generated for this study have been uploaded to Genome Sequencing Archive (GSA: CRA046744 and CRA046857), which is publicly accessible at https://ngdc.cncb.ac.cn/gsa.
