# Supplementary material for: Weissella confusa RY-1 ameliorates DSS-induced colitis by restoring intestinal barrier, modulating gut microbiota, and reprogramming microbial metabolism
Source: Front Microbiol. 2026 Aug 5;17:1896924. doi: 10.3389/fmicb.2026.1896924 (PMC13486278; doi:10.3389/fmicb.2026.1896924)
Supplement: Supplementary file 1 [file Data_sheet_1.pdf]

Supplementary Material

1 Supplementary Figures and Tables

1.1 Supplementary Figures

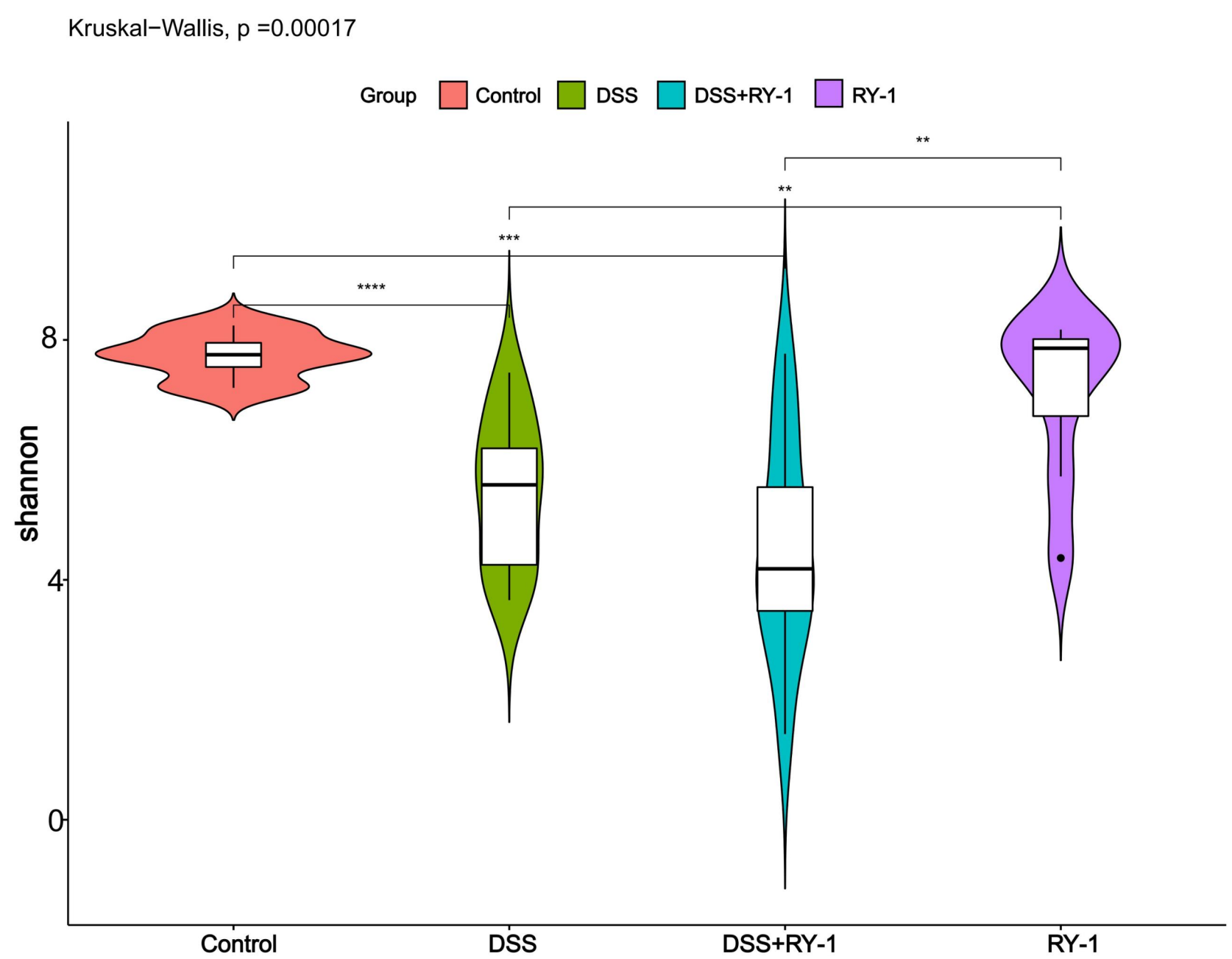

Supplementary Figure 1. Alpha diversity assessed by the Shannon index.
